# New pathways with high-sensitivity cardiac troponin testing at the point of care in the ambulance and primary care

**DOI:** 10.1093/ehjacc/zuaf157

**Published:** 2025-12-02

**Authors:** Tonje Rambøll Johannessen, Richard Body, Johannes Mair, Nicholas L Mills, Louise Cullen, the Study Group on Biomarkers of the ESC Association for Acute Cardiovascular Care, Bertil Lindahl, Jasper Boeddinghaus, Louise Cullen, Lori Daniels, Ola Hammarsten, Kurt Huber, Evangelos Giannitsis, Allan S Jaffe, Dorien M Kimenai, Konstantin A Krychtiuk, Martin Möckel, Christian Mueller, Matthias Thielmann, Kristian Thygesen, Johannes Mair, Nicholas L Mills

**Affiliations:** Department of General Practice, Institute of Health and Society, University of Oslo, Oslo, Norway; Department of Emergency General Practice, Oslo Accident and Emergency Outpatient Clinic, City of Oslo Health Agency, Oslo, Norway; Division of Cardiovascular Sciences, University of Manchester, Manchester, UK; Department of Internal Medicine III—Cardiology and Angiology, Medical University of Innsbruck, Innsbruck, Austria; BHF Centre for Cardiovascular Science, University of Edinburgh, SU.226 Chancellor’s Building, Royal Infirmary of Edinburgh, 49 Little France Crescent, Edinburgh EH16 4SU, UK; Emergency and Trauma Centre, Royal Brisbane and Women’s Hospital, Brisbane, Australia

## Abstract

**Graphical Abstract:**
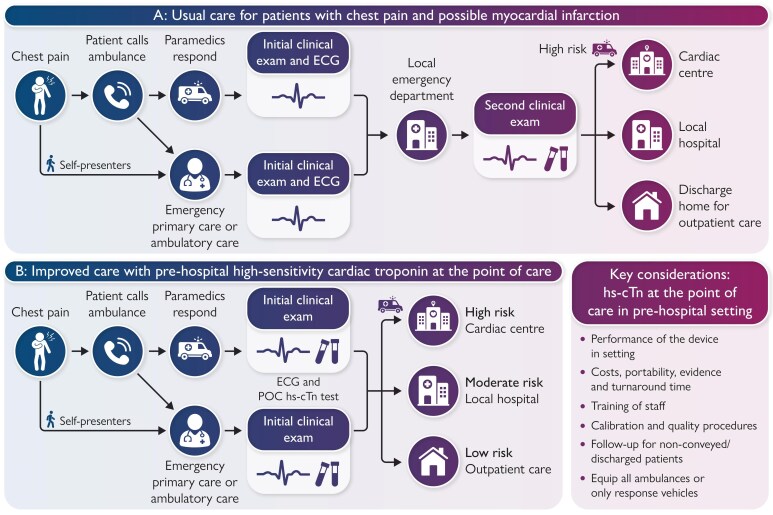
Improving care with pre-hospital high-sensitivity cardiac troponin testing at the point of care: pathways and considerations. (*A*) Usual care for patients with chest pain and possible myocardial infarction. (*B*) Improving care pathways with pre-hospital high-sensitivity cardiac troponin at the point of care in primary care and the ambulance.

Improving care with pre-hospital high-sensitivity cardiac troponin testing at the point of care: pathways and considerations. (*A*) Usual care for patients with chest pain and possible myocardial infarction. (*B*) Improving care pathways with pre-hospital high-sensitivity cardiac troponin at the point of care in primary care and the ambulance.

Chest pain is one of the most common reasons for emergency ambulance response. If myocardial infarction (MI) is suspected, patients are routinely conveyed to hospital for further investigations, which include one or more high-sensitivity cardiac troponin (hs-cTn) tests to confirm or exclude the diagnosis. In countries with a well-developed primary care network, chest pain is often initially assessed in a community setting. However, as no clinical decision tools can exclude MI without cardiac troponin testing, many patients are transferred to the hospital for further evaluation. With only 1 in 10 patients ultimately diagnosed with MI or an alternative serious condition requiring urgent treatment, the development of hs-cTn testing at the point of care (POC) has major potential to guide clinical decisions in a primary care and pre-hospital setting (*Graphical Abstract*).^1^

## Point-of-care testing in primary care

Chest pain assessment relies on clinical evaluation and an electrocardiogram. While some clinics have access to conventional POC cardiac troponin assays, it is challenging to undertake serial testing in this setting.^2^ In an observational cohort study, patients in Norway were assessed in emergency primary care setting with samples sent to a central laboratory for hs-cTnT testing. The majority were identified as low risk using the ESC 0/1-hour algorithm with a sensitivity of 98.4% and a negative predictive value (NPV) of 99.9%.^3^ The estimated cost saving was €1794 per patient by avoiding an unnecessary hospital admission.^4^ Access to hs-cTn POC testing and use of single-sample rule out pathways in this setting would further improve efficiency and could yield substantial cost reductions.

## Point-of-care testing in the ambulance

Point-of-care hs-cTn testing use by ambulance services involves paramedics performing the test at the scene, often in parallel with a 12-lead electrocardiogram during the initial assessment to improve the identification of appropriate patients for transfer. Though the time from symptom onset to testing is shorter in this setting than in an emergency department, hs-cTn testing has excellent sensitivity and NPV for MI when measured at least 3 h from symptom onset.^5^ A recent randomized trial found that prehospital testing with a non-high-sensitivity assay combined with a risk score significantly reduced costs (mean saving €717) with a similar incidence of major adverse cardiac events at 1 year.^6^

## Improving care pathways and overcoming logistical challenges

In both settings, POC hs-cTn testing could change care pathways in three ways. First, patients with high probability of MI could be conveyed directly to a cardiac centre for coronary angiography and revascularization, eliminating the need for secondary transfers and expediting specialist treatment. This would be especially beneficial in rural areas. Second, those with very low probability of MI or other serious conditions could be safely left at the scene or sent home from the clinic. Follow-up would need to be provided. Third, stable patients with low probability of MI could be assessed at a Same Day Emergency Care facility or referred to a cardiology outpatient clinic.

Implementation will require logistical challenges to be overcome. Cartridges may require storage in cold bags or refrigerators; cartridges must be at room temperature before testing; charging of the analyser must be guaranteed (power supply needed), paramedic and primary care staff training will be essential; follow-up arrangements must be clear; and when deployed by ambulance services, it is necessary to decide whether to deploy devices on all vehicles or have dedicated POC-enabled response vehicles.

If these challenges can be overcome, the opportunity to re-invent current care pathways with hs-cTn POC testing could provide more efficient, safe, patient-centred, and local care while reducing the burden on crowded EDs and stretched ambulance services.

## Data Availability

No data were generated or analysed for this manuscript.

## Appendix

**Members of the Study Group on Biomarkers of the ESC Association for Acute Cardiovascular Care include:**

Bertil Lindahl, MD^1^; Jasper Boeddinghaus, MD^2^; Louise Cullen, MD, PhD^3^; Lori Daniels, MD^4^; Ola Hammarsten, MD, PhD^5^; Kurt Huber, MD^6^; Evangelos Giannitsis, MD^7^; Allan S. Jaffe, MD^8^; Dorien M. Kimenai, PhD^9^; Konstantin A. Krychtiuk, MD, PhD^10^; Martin Möckel, MD^11^; Christian Mueller, MD^2^; Matthias Thielmann, MD^12^; Kristian Thygesen, MD^13^; Johannes Mair, MD^14^; and Nicholas L. Mills, MD, PhD^9^

^1^Department of Medical Sciences, University of Uppsala, Uppsala, Sweden

^2^Cardiovascular Research Institute Basel (CRIB) and Department of Cardiology, University Hospital Basel, University of Basel, Switzerland

^3^Emergency and Trauma Centre, Royal Brisbane and Women’s Hospital, Brisbane, Australia

^4^Department of Medicine, Sulpizio Cardiovascular Center, University of California, San Diego; La Jolla, CA, USA

^5^Department of Clinical Chemistry and Transfusion Medicine, University of Gothenburg, Gothenburg, Sweden

^6^Department of Medicine, Cardiology and Intensive Care Medicine, Wilhelminenhospital, and Sigmund Freud University, Medical School, Vienna, Austria

^7^Department of Cardiology, University Heidelberg, Germany

^8^Departments of Cardiology and Laboratory Medicine and Pathology, Mayo Clinic, Rochester, Minnesota, USA

^9^BHF Centre for Cardiovascular Science, University of Edinburgh, Edinburgh, UK

^10^Department of Internal Medicine II, Division of Cardiology, Medical University of Vienna, Vienna, Austria

^11^Division of Emergency Medicine, Charité-Universitätsmedizin Berlin, Germany

^12^Klinik für Thorax-und Kardiovaskuläre Chirurgie, Westdeutsches Herz- und Gefäßzentrum Essen, University of Duisburg-Essen, Germany

^13^Department of Cardiology, Aarhus University Hospital, Denmark

^14^Department of Internal Medicine III—Cardiology and Angiology, Innsbruck Medical University, Innsbruck, Austria
